# The dynamic interactions of policy instruments in China's community sports governance: a tripartite evolutionary game analysis

**DOI:** 10.3389/fpubh.2026.1801525

**Published:** 2026-03-31

**Authors:** Jiao Yuan, Yaxuan Liu, Xianglin Luo

**Affiliations:** School of Physical Education, Hunan Normal University, Changsha, China

**Keywords:** collaborative governance, community sports, digital empowerment, evolutionary game theory, public health

## Abstract

Community sports governance critically shapes public health by affecting physical activity and chronic disease prevention. In China, shifting toward collaborative governance involving local governments, communities, and sports organizations is vital for optimizing public health resources, yet the strategic dynamics among these stakeholders remain poorly understood. This study employs a tripartite evolutionary game model to analyze their interactions, incorporating three policy instruments: fiscal incentives (α), performance pressure (β), and digital empowerment (γ). Numerical simulations reveal that intermediate subsidy levels and relaxed performance metrics often induce persistent strategic oscillations, undermining service stability. Conversely, strong administrative oversight (low β) and, more effectively, robust digital empowerment (high γ) significantly stabilize cooperation. A “Technology-Driven” strategy emerges as the most efficient pathway to a stable, fully cooperative equilibrium, ensuring reliable sports service delivery. The study concludes that effective governance requires a shift from subsidy reliance to frameworks centered on digital enablement and intelligent accountability. By lowering coordination costs and aligning stakeholder incentives, such an approach can foster a sustainable collaborative ecosystem that promotes physical activity and improves population health outcomes.

## 1 Introduction

Community sports governance, as a pivotal component of China's national governance modernization initiative, has garnered substantial attention from both policymakers and researchers. Following the implementation of the “Healthy China” strategy and the strategic objective of establishing a “sports power,” community sports have progressively emerged as a critical domain for advancing public health outcomes, strengthening social cohesion, and facilitating the high-quality development of the sports industry. From a public health perspective, the governance failures in community sports can translate into preventable disease burdens and health inequities. In China, where rapid urbanization has been accompanied by declining physical activity (1) and rising obesity rates (2), the stakes of governance failure extend beyond administrative inefficiency to encompass population health crises. Indeed, physical inactivity is a primary driver of non-communicable disease

prevalence in China (3). Therefore, understanding the policy mechanisms that enable or hinder collaborative governance in community sports is not merely an administrative concern but a pressing public health imperative. Empirical evidence demonstrates that community sports provision significantly contributes to social inclusion and public health improvement, particularly through the construction of public sports facilities (4), which have been shown to increase participation in community sports and decrease health risks.

Nevertheless, contemporary community sports governance in China confronts substantial systemic challenges, including “administrative dependence,” “organizational suspension,” and the phenomenon of “high-level enthusiasm coupled with grassroots disengagement,” which collectively illuminate the intricate multi-stakeholder dynamics and the inherent constraints of prevailing policy frameworks.

As policies at various levels continue to emphasize sports development, the interactive relationships among local government, communities, and sports organizations have gradually evolved into a complex dynamic game system. Internationally, sports governance has undergone a fundamental paradigm shift, moving away from hierarchical, government-led models toward networked, polycentric collaborative frameworks (5). However, this transformation is not universally applicable. The “Western model” of bottom-up, civil-society-driven sports development is incompatible with China's institutional context. In fact, China's sports policy implementation is plagued by “fragmentation” in vertical authority structures and “disconnection” in horizontal coordination mechanisms, manifesting as systematic failures in aligning central directives with local execution capacities (6). Existing governance frameworks are built on implicit assumptions of autonomous civil society actors and market-driven resource allocation. However, in China, community organizations often operate under a condition of administrative embedding, while sports institutions must navigate hybrid logics that blend market and state imperatives (7). Within this context, the design and implementation of policy instrument mixes for community sports governance become critical not only for enhancing administrative efficacy but also for advancing public health outcomes.

Existing research on community sports governance and policy intervention can be broadly categorized into two interconnected streams: policy instrument analysis and collaborative governance frameworks. Research on policy instruments has effectively explored how specific interventions shape stakeholder behavior in sports and public health contexts (8). Empirical work in China confirms correlations between government support, community facilities, and physical activity participation (9), as well as the mediating role of governance structures (10). Seminal studies have systematically analyzed instrument selection and their role in collaborative governance (11, 12).

However, these work exhibits critical limitations in the Chinese context. First, existing studies analyze instruments in isolation, neglecting the dynamic complementarity or substitution effects within a policy mix (13, 14). Specifically, they fail to model the critical substitution and complementarity effects between different policy instruments, such as the potential for market-based incentives and informational tools to reduce the reliance on administrative pressure. Second, documented policy-practice gaps, such as structural misalignments termed “organizational suspension” (15) or passive policy diffusion under dual central-local pressures (16), remain theoretically unintegrated. It fails to model how specific instruments reconfigure the strategic calculations of heterogenous actors operating within China's unique asymmetric power structures. Third, policy impact is frequently assumed to be proportional and monotonic. While scholars like (17) emphasize policy coherence and instrument combinations, they overlook potential threshold effects and non-linear transitions that could lead to suboptimal equilibrium. These limitations underscore the need for an analytical approach that can dynamically model strategic interactions within policy mixes.

Research on multi-stakeholder collaborative governance provides crucial frameworks, emphasizing consensus, shared goals, and institutional design (18). While these models are essential for understanding polycentric systems (19), their empirical application within the Chinese context reveals a profound theoretical paradox where the underlying assumption of symmetrical power and resource distribution proves inadequate in the face of imbalanced governance environments (20). In China's community sports sector, the relationship between the government, resource-constrained community organizations, and market-driven sports entities is inherently asymmetric (21). The government possesses unique administrative power and political incentives that fundamentally dictate the strategic choices of the other two actors. Existing models, including those on policy implementation networks (22), fail to explicitly incorporate this institutional asymmetry into the payoff structure, thus limiting their ability to explain phenomena like “administrative dependence” and “organizational suspension” that characterize the Chinese grassroots level.

Understanding how different policy tools shape the tripartite interactions among local government, community, and sports organizations—and how these interactions evolve over time—requires an analytical approach capable of capturing strategic adaptability and interdependent decision-making. Hence, evolutionary game approach offers a fitting lens to examine how policy instruments can be calibrated to foster cooperative governance, and ultimately promote public health through sustained community sports engagement. The application of Evolutionary Game Theory (EGT) has proven robust for modeling strategic evolution under bounded rationality. Recent EGT studies in Chinese public administration have explored multi-agent interactions in areas such as public health (23) and environmental management (24). More recently, tripartite EGT models have been applied to sports data rights governance (25), smart older adults platform governance (26), data security governance with cross-level government supervision (27), and multi-stakeholder AI governance with dynamic reward-penalty mechanisms (28), all demonstrating that digital empowerment and dynamic accountability mechanisms are essential for stabilizing multi-agent cooperative equilibria in Chinese public service governance. Nonetheless, there is little research on the in-depth analysis of the internal mechanisms of community sports governance and the interaction among multiple stakeholders. Moreover, the government's behavior is heavily influenced by the “pressure-type system”—a mechanism where political accountability and career advancement are directly tied to performance metrics imposed by superior authorities (29). By failing to explicitly integrate this political performance pressure into the government's payoff function, simplifying the true behavioral logic of the most dominant actor, thus limiting their explanatory power and policy relevance for addressing China's distinctive governance challenges.

Therefore, this study systematically constructs a tripartite evolutionary game model that explicitly incorporates policy instrument mix effects, institutional asymmetry in China's governance structure, and context-specific constraints of the “pressure-type system.” Unlike existing models that treat policy instruments as isolated variables, we incorporate fiscal incentives, performance regulations, and technological empowerment into the model, enabling analysis of non-linear synergies, threshold effects, and potential policy conflicts. This advances beyond Lindsey and Darby (17)'s call for policy coherence by providing a formal mechanism to identify optimal instrument combinations and diagnose counterproductive interactions. Meanwhile, we analyze the non-linear synergistic and substitution effects between fiscal incentives, performance pressure, and technological empowerment, identifying the critical thresholds for effective policy combination. By integrating political performance pressure into the government's payoff function, we offer a novel analytical framework for understanding how the “pressure-type system” shapes the strategic evolution of community sports governance in China.

The remainder of this paper is organized as follows: Section 2 presents the theoretical model and analytical framework; Section 3 provides the results of theoretical analysis and numerical analysis; Section 4 discusses the theoretical implications, policy recommendations, and limitations. Section 5 concludes with a summary of the key findings.

## 2 Model construction and analysis

### 2.1 Model assumptions and parameter settings

To provide empirical evidence for the behavior assumptions of game participants, this study references the widely documented “Community Sports Home” initiative in Jiaxing, Zhejiang Province—a nationally recognized model for technology-mediated, multi-agent community governance. The initiative's operational data and case summaries provide direct real-world analogues for the model's abstract strategy sets. For instance, from providing tailored services, co-creating programs to offering standardized, low-investment activities, informs the “Professional Engagement/Conservative Operation” strategy dichotomy for sports organizations. The initiative's operational mechanisms—such as digital platforms reducing participation costs and social incentive systems enhancing benefits—provide empirical basis for calibrating key payoff parameters (e.g., cost sensitivity, social benefit).

This study constructs a tripartite evolutionary game model involving local government departments (G), community organizations (C), and sports organizations (S). The key assumptions and parameter settings are as follows:

**Assumption 1**: Let *x* denote the probability that the local government adopts the “Proactive Support” strategy (denoted by “A”), and 1−*x* the probability it adopts the “Passive Observance” strategy (denoted by “P”).

If “Proactive Support” is chosen: The government commits dedicated fiscal resources, formulates supportive policies, and initiates collaborative platforms, thereby incurring a fixed administrative and financial cost *C*_*G*_. In return, it receives a base level of political performance benefit *R*_*G*_ (e.g., fulfilling higher-level targets for sports facility development, reflecting improved public health and social cohesion), regardless of other participants' actions. Meanwhile, the government will provide cost subsidies to communities and sports organizations undertaking the same initiative at a certain subsidy rate α. If both the community and the sports organization respond actively and form an effective collaboration, the government garners an additional synergistic governance benefit Δ*R*_*G*_ (e.g., the project being recognized as a provincial or national model case, receiving special commendations, significantly enhancing public satisfaction and governmental credibility).

If “Passive Observance” is chosen: The government refrains from active resource allocation, maintaining only routine supervision. In this case, if the community and sports organization spontaneously engage actively and achieve results, the government can “free-ride,” appropriating a share of the performance outcomes, quantified as β*R*_*G*_ (where 0 ≤ β ≤ 1 represents the appropriation ratio, possibly realized by reporting subordinate achievements). However, choosing this strategy inherently exposes the government to potential accountability risks and reputational loss *L*_*G*_ (e.g., negative media exposure, criticism from superior inspections, increased public complaints), irrespective of other players' strategies. In fact, *L*_*G*_ reflects the cost of political accountability or the penalty for poor performance imposed by the superior government due to governance failures within China's centralized political system and decentralized fiscal structure (30). Moreover, if all three participants remain inactive, its payoff is normalized to 0.

**Assumption 2**: Let *y* denote the probability that the community adopts the “Collaborative Mobilization” strategy (denoted by “A”), and 1−*y* the probability it adopts the “Routine Maintenance” strategy (denoted by “P”).

If “Collaborative Mobilization” is chosen: Community organizations operate under significant resource constraints and are highly dependent on external funding and local legitimacy (31). The community organization must invest substantial effort in resident outreach, venue coordination, activity organization, and on-site management, incurring a coordination cost *C*_*C*_. This cost is mitigated by the level of governmental support: both fiscal subsidies (at rate α) and technological empowerment (at rate γ, e.g., provision of digital management platforms) reduce the community's effective burden to (1−α−γ)*C*_*C*_ where α+γ ≤ 1. It is worth noting that the model explicitly encompasses the scenario where only technological empowerment is provided without fiscal subsidies. In practice, “Technology-Only” governance models are emerging in digitally mature regions, demonstrating the substitutive or complementary role of digital empowerment to fiscal incentives (32). For instance, the “Zhejiang Sports Code” provides a unified digital interface for community sports without necessarily requiring continuous fiscal subsidies for every activity. In our model, this corresponds to a scenario where α = 0, and the community's coordination burden is reduced to (1−γ)*C*_*C*_ through digital efficiency gains. This reflects the government's investment in public digital infrastructure that lowers transaction costs for community organizations. Its base benefit *R*_*C*_ derives from enhanced community vitality and resident satisfaction. Effective collaboration with the government and sports organization yields an additional synergistic benefit Δ*R*_*C*_ (e.g., access to higher-quality public resources, establishment of branded community events).

If “Routine Maintenance” is chosen: The community provides only basic public services without organizing additional sports activities. Choosing inaction results in a loss of credibility and satisfaction *L*_*C*_, regardless of other participants' strategies. Here, *L*_*C*_ represents the loss of organizational legitimacy and social capital resulting from failing to meet community needs, which is critical for their long-term survival and resource mobilization (19). The payoff is 0 if all parties are inactive.

**Assumption 3**: Let *z* denote the probability that the sports organization adopts the “Professional Engagement” strategy (denoted by “A”), and 1−*z* the probability it adopts the “Conservative Operation” strategy (denoted by “P”).

If “Professional Engagement” is chosen: Sports organizations are primarily driven by market incentives and the strategic goal of industry expansion, heavily influenced by national policy guidance (33). The sports organizations invest in professional coaches, program development, equipment, and marketing, incurring an operational cost *C*_*S*_. If it secures government procurement contracts or subsidies (at rate α), its effective cost reduces to (1−α)*C*_*S*_. Its base benefit *R*_*S*_ primarily comes from service fees, brand exposure, and market share. Deep collaboration with the government and community generates a synergistic benefit Δ*R*_*S*_(e.g., securing long-term stable government contracts, embedding services within the community to build user loyalty, enhancing brand reputation).

If “Conservative Operation” is chosen: The sports organizations conduct only routine commercial activities without proactively seeking deep collaborative projects with the government and community. This choice leads to a loss of developmental opportunity and brand stagnation *L*_*S*_, irrespective of others' actions. Here, *L*_*S*_ denotes the opportunity cost of missing out on government-supported market expansion and potential contracts, which are crucial for growth in China's policy-driven sports sector (34). The payoff is 0 under universal inaction.

**Assumption 4**: The model incorporates a policy instrument mix designed to address the collective action problem. (1) Fiscal incentive [α∈[0, 1]]: The subsidy rate α acts as a market-based instrument by directly reducing the active participation costs (*C*_*C*_, *C*_*S*_) for the community and sports organizations, a common strategy to overcome initial investment barriers (35). (2) Political incentive [β∈[0, 1]]: The performance evaluation discount coefficient β quantifies the degree of political benefit retention for local governments under superior oversight, where a lower β indicates stricter evaluation criteria. Although formal evaluations are conducted ex-post (after governance outcomes are observable), the anticipation of accountability creates “constructive pressure” (29, 36). This anticipation transforms the ex-post evaluation into an ex-ante incentive mechanism: a low β significantly reduces the expected political payoff from passive governance to β*R*_*G*_ rather than the full *R*_*G*_, thereby creating forward-looking behavioral incentives by penalizing inaction in advance. Thus, the evaluation's timing after the decision does not diminish its role as constructive pressure; instead, the pre-decision expectation of accountability aligns local actions with superior goals through anticipated rewards or penalties. (3) Technology empowerment [γ∈[0, 1]]: The information platform effectiveness γ acts as an informational instrument, reducing the coordination cost (*C*_*C*_) for the community, reflecting the role of digital governance in lowering transaction costs and mitigating information asymmetry. In this study, the digital governance infrastructure associated with γ operationalizes what we term “intelligent accountability”—defined as an automatic performance feedback mechanism that leverages real-time data streams from sports-app participation records, community check-in logs, and facility-usage sensors to generate transparent, continuous performance signals for all three stakeholders. This mechanism operationally links the theoretical parameter γ to the practical governance innovation of algorithmically enforced transparency (10, 37). Here, γ = 0 means the information platform provides zero cost reduction for community coordination (no technical empowerment). γ = 1 means the information platform enables the complete elimination of coordination costs (full technical empowerment).

Based on these assumptions, Table 1 further describes the relevant notations. Moreover, the complete payoff matrix for each stakeholder under different strategy combinations is presented in Table 2.

**Table 1:** Model notations.

Notation	Explanation
*R*_*i*_, *i*∈{*G, C, S*}	The base benefit for participant *i* when choosing “A”
*C*_*i*_	The operational cost for participant *i* when choosing “A”
*L*_*i*_	The reputational, accountability, or opportunity losses for participant *i* when choosing “P”
Δ*R*_*i*_	The synergistic benefits for participant *i* when all three participants choose “A”
α	Subsidy rate of special funds provided by local government
β	Performance evaluation discount coefficient
γ	Efficiency of information platform in reducing the cost of community coordination

**Table 2:** Payoff matrix for tripartite evolutionary game.

Strategy profile	Local government (G)	Community (C)	Sports Org. (S)
(A,A,A)	*R*_*G*_+Δ*R*_*G*_−*C*_*G*_−α(*C*_*C*_+*C*_*S*_)	*R*_*C*_+Δ*R*_*C*_−(1−α−γ)*C*_*C*_	*R*_*S*_+Δ*R*_*S*_−(1−α)*C*_*S*_
(A,A,P)	*R*_*G*_−*C*_*G*_−α*C*_*C*_	*R*_*C*_−(1−α−γ)*C*_*C*_	−*L*_*S*_
(A,P,A)	*R*_*G*_−*C*_*G*_−α*C*_*S*_	−*L*_*C*_	*R*_*S*_−(1−α)*C*_*S*_
(A,P,P)	*R*_*G*_−*C*_*G*_	−*L*_*C*_	−*L*_*S*_
(P,A,A)	β*R*_*G*_−*L*_*G*_	*R*_*C*_−*C*_*C*_	*R*_*S*_−*C*_*S*_
(P,A,P)	β*R*_*G*_−*L*_*G*_	*R*_*C*_−*C*_*C*_	−*L*_*S*_
(P,P,A)	β*R*_*G*_−*L*_*G*_	−*L*_*C*_	*R*_*S*_−*C*_*S*_
(P,P,P)	0	0	0

The expected payoffs for different strategy combinations can be calculated as follows:

When local government departments choose “A”, their expected payoff is:


$$\begin{array}{c} E_{GA}=yz(R_{G}+\text{Δ}R_{G}-C_{G}-\alpha \left(C_{C}+C_{S}\right)\\ +y(1-z)\left(R_{G}-C_{G}-\alpha C_{C}\right)\\ +(1-y)z\left(R_{G}-C_{G}-\alpha C_{S}\right)+(1-y)(1-z)\left(R_{G}-C_{G}\right)\\ =R_{G}-C_{G}+yz\text{Δ}R_{G}-\alpha \left(yC_{C}+zC_{S}\right) \end{array}$$ (1)

When communities choose “A”, their expected payoff is:


$$\begin{array}{c} E_{GP}=yz\left(\beta R_{G}-L_{G}\right)+y(1-z)\left(\beta R_{G}-L_{G}\right)\\ +(1-y)z\left(\beta R_{G}-L_{G}\right)+(1-y)\\ =(y+z-yz)\left(\beta R_{G}-L_{G}\right) \end{array}$$ (2)

Based on Equations 1 and 2, we can obtain the following replicator dynamics equation for local government departments (see Equation 3).


$$\begin{array}{cc} F\left(x\right) & =\frac{dx}{dt}=x\left(1-x\right)\left(E_{GA}-E_{GP}\right)=x\left(1-x\right)H\left(z\right) \end{array}$$ (3)

where *H*(*z*) = *R*_*G*_−*C*_*G*_+*yzΔR*_*G*_−α(*yC*_*C*_+*zC*_*S*_)−(*y*+*z*−*yz*)(β*R*_*G*_−*L*_*G*_).

Similarly, we can derive the expected payoffs and replicator dynamics equations for communities and sports organizations as follows (see Equations 4 and 5).


$$\begin{array}{cc} F\left(y\right) & =\frac{dy}{dt}=y\left(1-y\right)H\left(x\right) \end{array}$$ (4)

$$\begin{array}{cc} F\left(z\right) & =\frac{dz}{dt}=z\left(1-z\right)H\left(y\right) \end{array}$$ (5)

where *H*(*x*) = *R*_*C*_−*C*_*C*_+*x*(α+γ)*C*_*C*_+*xzΔR*_*C*_+(*x*+*z*−*xz*)*L*_*C*_, *H*(*y*) = *R*_*S*_−*C*_*S*_+*xαC*_*S*_+*xyΔR*_*S*_+(*x*+*y*−*xy*)*L*_*S*_.

From the stability theorem of the differential equation, it follows that the probability of the local government choosing “A” is in a steady state must satisfy: *F*(*x*) = 0 and $\frac{dF\left(x\right)}{dx}<0$. If $z=\frac{y\left(\alpha C_{S}+\beta R_{G}-L_{G}\right)+C_{G}-R_{G}}{y\Delta R_{G}-\left(1-y\right)\left(\beta R_{G}-L_{G}\right)-\alpha C_{C}}=z^{*}$, then $\frac{dF\left(x\right)}{dx}=0$. Since $\frac{dH\left(z\right)}{dz}=y\Delta R_{G}-\left(1-y\right)\left(\beta R_{G}-L_{G}\right)-\alpha C_{C}$, if *yΔR*_*G*_ < (1−*y*)(β*R*_*G*_−*L*_*G*_)+α*C*_*C*_, then *H*(*z*) is a decreasing function with respect to *z*. Thus, when *z*<z^*^, then $\frac{dF\left(x\right)}{dx}{|}_{x=1}<0$, at this time, *x* = 1 is the evolutionary stabilization strategy for the local government. Conversely when *z*>z^*^, then $\frac{dF\left(x\right)}{dx}{|}_{x=0}<0$, thus *x* = 0 is the evolutionary stabilization strategy for the local government. Similarly, if *yΔR*_*G*_>(1−*y*)(β*R*_*G*_−*L*_*G*_)+α*C*_*C*_, then *H*(*z*) is a increasing function with respect to *y*. Thus, when *z*<z^*^, then $\frac{dF\left(x\right)}{dx}{|}_{x=0}<0$, at this time, *x* = 0 is the evolutionary stabilization strategy for the local government. Conversely when *z*>z^*^, then $\frac{dF\left(x\right)}{dx}{|}_{x=1}<0$, thus *x* = 1 is the evolutionary stabilization strategy for the local government.

Similarly, for community, if $x=\frac{C_{C}-R_{C}-zL_{C}}{\left(\alpha +\gamma \right)C_{C}+\left(1-z\right)L_{C}+z\Delta R_{C}}=x^{*}$, then $\frac{dF\left(y\right)}{dy}=0$. Since $\frac{dH\left(x\right)}{dx}=\left(\alpha +\gamma \right)C_{C}+\left(1-z\right)L_{C}+z\Delta R_{C}>0$, then *H*(*x*) is a increasing function with respect to *x*. Thus, when *x*<x^*^, then $\frac{dF\left(y\right)}{dy}{|}_{y=0}<0$, at this time, *y* = 0 is the evolutionary stabilization strategy for the community. Conversely when *x*>x^*^, then $\frac{dF\left(y\right)}{dy}{|}_{y=1}<0$, thus *y* = 1 is the evolutionary stabilization strategy for the community.

For sports organizations, if $y=\frac{C_{S}-R_{S}-x\left(\alpha C_{S}+L_{S}\right)}{x\Delta R_{S}+\left(1-x\right)L_{S}}=y^{*}$, then $\frac{dF\left(z\right)}{dz}=0$. Since $\frac{dH\left(y\right)}{dy}=x\Delta R_{S}+\left(1-x\right)L_{S}>0$, then *H*(*y*) is a increasing function with respect to *y*. Thus, when *y*<y^*^, then $\frac{dF\left(z\right)}{dz}{|}_{z=0}<0$, at this time, *z* = 0 is the evolutionary stabilization strategy for the sports organizations. Conversely when *y*>y^*^, then $\frac{dF\left(z\right)}{dz}{|}_{z=1}<0$, thus *z* = 1 is the evolutionary stabilization strategy for the sports organizations.

### 2.2 Equilibrium point and stability analysis

The equilibrium solution of the tripartite evolutionary game is a strict Nash equilibrium (38), therefore, we only need to discuss the asymptotic stability of the eight equilibrium points, i.e., *E*_1_(1, 1, 1), *E*_2_(1, 1, 0), *E*_3_(1, 0, 1), *E*_4_(1, 0, 0), *E*_5_(0, 1, 1), *E*_6_(0, 1, 0), *E*_7_(0, 0, 1) and *E*_8_(0, 0, 0). The stability of the equilibrium point in the evolutionary game model was obtained by analyzing the Jacobi matrix (see Equation 6). System stability was analyzed by solving the eigenvalues of the Jacobi matrix corresponding to each equilibrium point (see Tables 3, 4).


$$\begin{array}{c} J=\left(\begin{array}{ccc} \frac{\partial F\left(x\right)}{\partial x} & \frac{\partial F\left(x\right)}{\partial y} & \frac{\partial F\left(x\right)}{\partial z}\\ \frac{\partial F\left(y\right)}{\partial x} & \frac{\partial F\left(y\right)}{\partial y} & \frac{\partial F\left(y\right)}{\partial z}\\ \frac{\partial F\left(z\right)}{\partial x} & \frac{\partial F\left(z\right)}{\partial y} & \frac{\partial F\left(z\right)}{\partial z} \end{array}\right) \end{array}$$ (6)

**Table 3:** Eigenvalues of Jacobi matrix.

Points	λ_1_	λ_2_	λ_3_
*E*_1_(1, 1, 1)	−((1−β)*R*_*G*_+Δ*R*_*G*_+*L*_*G*_−α(*C*_*C*_+*C*_*S*_)−*C*_*G*_)	−(*R*_*C*_+Δ*R*_*C*_+*L*_*C*_−(1−α−γ)*C*_*C*_)	−(*R*_*S*_+Δ*R*_*S*_+*L*_*S*_−(1−α)*C*_*S*_)
*E*_2_(1, 1, 0)	−(*R*_*G*_−*C*_*G*_−α*C*_*C*_−(β*R*_*G*_−*L*_*G*_))	−(*R*_*C*_−(1−α−γ)*C*_*C*_+*L*_*C*_)	*R*_*S*_−(1−α)*C*_*S*_+Δ*R*_*S*_+*L*_*S*_
*E*_3_(1, 0, 1)	−(*R*_*G*_−*C*_*G*_−α*C*_*S*_−(β*R*_*G*_−*L*_*G*_))	*R*_*C*_−(1−α−γ)*C*_*C*_+Δ*R*_*C*_+*L*_*C*_	−(*R*_*S*_−(1−α)*C*_*S*_+*L*_*S*_)
*E*_4_(1, 0, 0)	−(*R*_*G*_−*C*_*G*_)	*R*_*C*_−(1−α−γ)*C*_*C*_+*L*_*C*_	*R*_*S*_−(1−α)*C*_*S*_+*L*_*S*_
*E*_5_(0, 1, 1)	(1−β)*R*_*G*_+Δ*R*_*G*_+*L*_*G*_−α(*C*_*C*_+*C*_*S*_)−*C*_*G*_	−(*R*_*C*_−*C*_*C*_+*L*_*C*_)	−(*R*_*S*_−*C*_*S*_+*L*_*S*_)
*E*_6_(0, 1, 0)	*R*_*G*_−*C*_*G*_−α*C*_*C*_−(β*R*_*G*_−*L*_*G*_)	−(*R*_*C*_−*C*_*C*_)	*R*_*S*_−*C*_*S*_+*L*_*S*_
*E*_7_(0, 0, 1)	*R*_*G*_−*C*_*G*_−α*C*_*S*_−(β*R*_*G*_−*L*_*G*_)	*R*_*C*_−*C*_*C*_+*L*_*C*_	−(*R*_*S*_−*C*_*S*_)
*E*_8_(0, 0, 0)	*R*_*G*_−*C*_*G*_	*R*_*C*_−*C*_*C*_	*R*_*S*_−*C*_*S*_

**Table 4:** Stability conditions of equilibrium point.

Points	Stability conditions
*E*_1_(1, 1, 1)	(1−β)*R*_*G*_+Δ*R*_*G*_+*L*_*G*_>α(*C*_*C*_+*C*_*S*_)+*C*_*G*_, *R*_*C*_+Δ*R*_*C*_+*L*_*C*_>(1−α−γ)*C*_*C*_ and *R*_*S*_+Δ*R*_*S*_+*L*_*S*_>(1−α)*C*_*S*_
*E*_2_(1, 1, 0)	(1−β)*R*_*G*_+*L*_*G*_>α*C*_*C*_+*C*_*G*_, *R*_*C*_+*L*_*C*_>(1−α−γ)*C*_*C*_ and *R*_*S*_+*L*_*S*_+Δ*R*_*S*_ < (1−α)*C*_*S*_
*E*_3_(1, 0, 1)	(1−β)*R*_*G*_+*L*_*G*_>α*C*_*S*_+*C*_*G*_, *R*_*C*_+Δ*R*_*C*_+*L*_*C*_ < (1−α−γ)*C*_*C*_ and *R*_*S*_+*L*_*S*_>(1−α)*C*_*S*_
*E*_4_(1, 0, 0)	*R*_*G*_>*C*_*G*_, *R*_*C*_+*L*_*C*_ < (1−α−γ)*C*_*C*_ and *R*_*S*_+*L*_*S*_ < (1−α)*C*_*S*_
*E*_5_(0, 1, 1)	(1−β)*R*_*G*_+Δ*R*_*G*_+*L*_*G*_ < α(*C*_*C*_+*C*_*S*_)+*C*_*G*_, *R*_*C*_+*L*_*C*_>*C*_*C*_ and *R*_*S*_+*L*_*S*_>*C*_*S*_
*E*_6_(0, 1, 0)	(1−β)*R*_*G*_+*L*_*G*_ < α*C*_*C*_+*C*_*G*_, *R*_*C*_>*C*_*C*_ and *R*_*S*_+*L*_*S*_<*C*_*S*_
*E*_7_(0, 0, 1)	(1−β)*R*_*G*_+*L*_*G*_ < α*C*_*S*_+*C*_*G*_, *R*_*C*_+*L*_*C*_<*C*_*C*_ and *R*_*S*_>*C*_*S*_
*E*_8_(0, 0, 0)	*R*_*G*_<*C*_*G*_, *R*_*C*_<*C*_*C*_ and *R*_*S*_<*C*_*S*_

The stability conditions of the Jacobi matrix eigenvalues (Table 4) reveals fundamental structural relationships between policy parameters and system equilibrium. Eight potential equilibrium points demonstrate distinct stability conditions, with the optimal collaborative state *E*_1_(1, 1, 1) requiring simultaneous satisfaction of three synergistic thresholds: (1−β)*R*_*G*_+Δ*R*_*G*_+*L*_*G*_>α(*C*_*C*_+*C*_*S*_)+*C*_*G*_, *R*_*C*_+Δ*R*_*C*_+*L*_*C*_>(1−α−γ)*C*_*C*_, and *R*_*S*_+Δ*R*_*S*_+*L*_*S*_>(1−α)*C*_*S*_. These conditions illuminate how each policy instrument creates distinct incentive channels. Fiscal subsidies (α) reduce net collaboration costs across all three agents, lowering the thresholds for achieving evolutionary stable strategies (ESS) and serving as a universal cost-sharing mechanism. Performance evaluation (β) specifically targets the local government by diminishing its relative passive gains, thereby elevating its ESS threshold while motivating active supervision of community and sports organizations. Information platforms (γ) exclusively empower community organizations by reducing their operational burdens, embodying the principle of “technological empowerment” rather than direct financial transfer. The alternative equilibrium *E*_8_(0, 0, 0) emerges under conditions of universal cost-benefit imbalance (*R*_*G*_<*C*_*G*_, *R*_*C*_<*C*_*C*_, *R*_*S*_<*C*_*S*_), representing systemic coordination failure. Intermediate equilibria (*E*_2_ through *E*_7_) reveal how partial cooperation patterns depend on specific parameter configurations, with boundary conditions illustrating threshold effects where marginal changes in policy parameters can trigger discontinuous shifts in equilibrium outcomes. Collectively, α and γ constitute positive incentive mechanisms that reduce participation barriers, while β establishes regulatory discipline through performance accountability.

**Figure 1:**
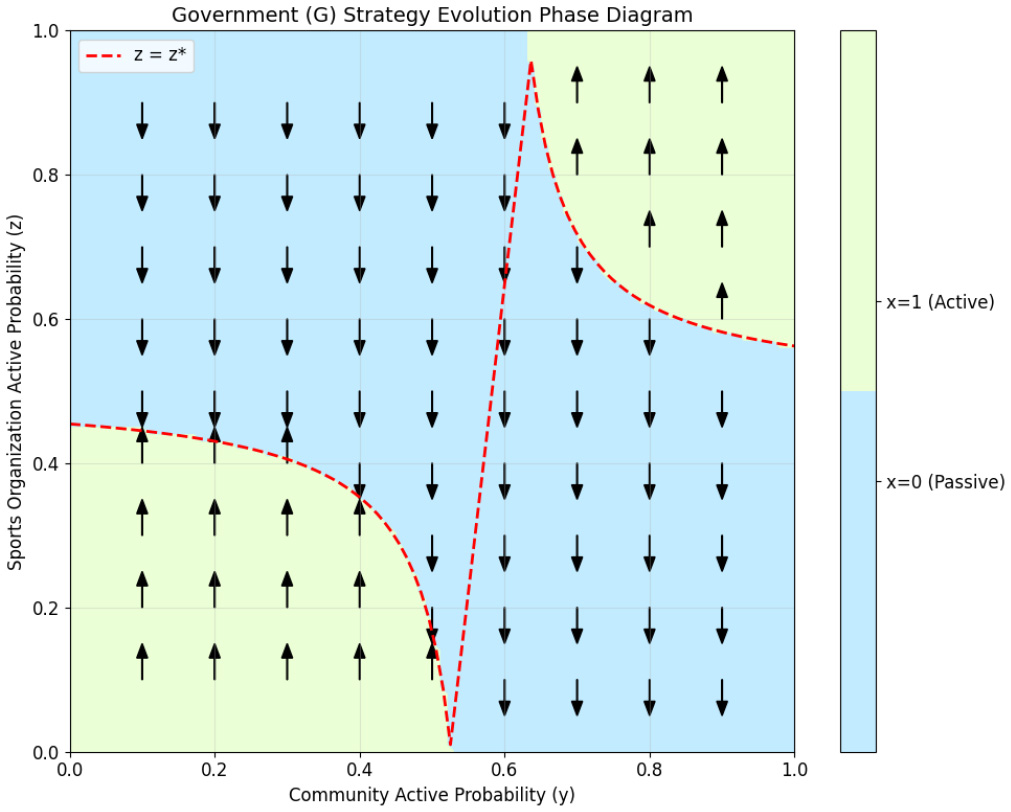
The evolutionary phase diagram of the local government.

**Figure 2:**
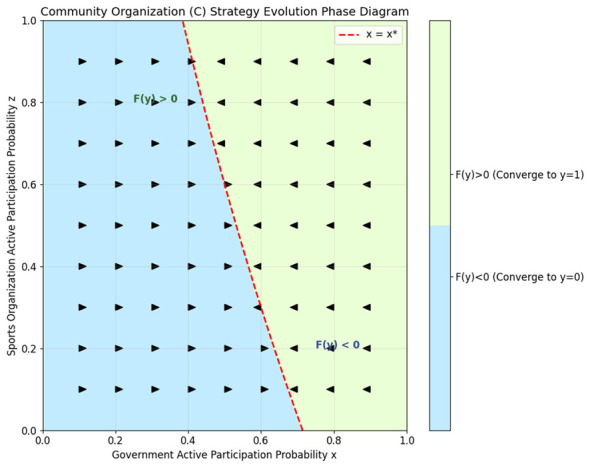
Evolutionary phase diagram of the community organizations.

**Figure 3:**
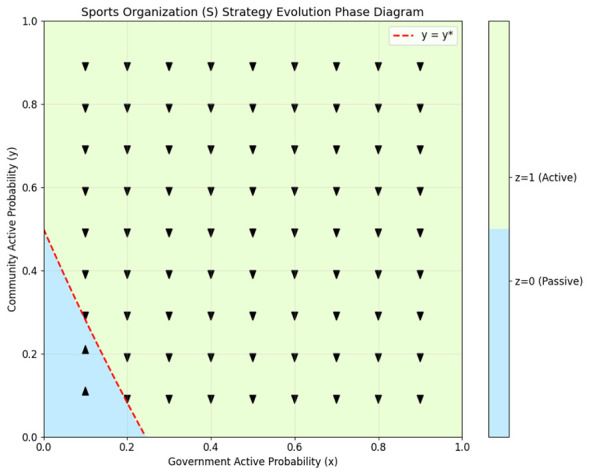
Evolutionary phase diagram of the sports organizations.

The two-dimensional projection phase diagrams of the three participants are shown in Figures 1–3, clearly illustrating their strategy evolution trend and the evolutionary stable strategy boundary.

From Figures 1–3, the evolutionary phase diagrams reveal a hierarchical interdependence among stakeholders' strategic choices. The government's adoption of active governance is contingent upon sports organizations' participation exceeding a critical threshold z^*^, which itself varies with community engagement levels. Community organizations exhibit threshold-responsive behavior, converging to active cooperation only when governmental involvement surpasses x^*^, with sports organizations' participation moderating this threshold. Sports organizations, in turn, require community participation above y^*^ to sustain active strategies, a threshold lowered by governmental support. This cascading dependency structure demonstrates that policy interventions targeting any single actor can generate multiplier effects across the system.

## 3 Numerical analysis

**Figure 4:**
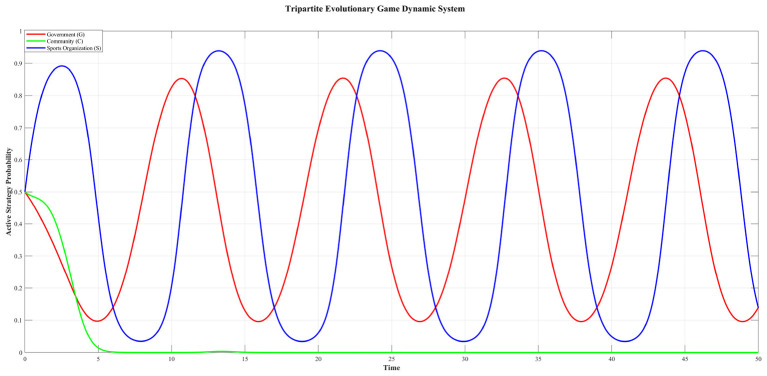
Evolutionary phase diagram of the sports organizations.

In order to verify the validity of the evolutionary stability analysis, we conduct a numerical analysis of the model. The parameter values for the numerical simulation are calibrated with reference to empirical data from the “Community Sports Home” initiative in Jiaxing, Zhejiang Province. Considering direct monetary quantification is challenging, the initial values of several parameters—which are difficult to determine through direct assignment from realistic data—are standardized on a relative scale (e.g., from 0 to 10) or as rates (between 0 and 1), informed by the operational logic and observed outcomes of the case. Although some initial parameter values are not supported by empirical data, they can be used to analyze and explore mechanisms that promote the stability of community sports governance. Moreover, considering the reality in many Chinese communities, where coordination failures and free-riding behaviors prevail, making it essential to understand the conditions required to transition from this situation toward active collaborative governance. We set the following parameter values for numerical simulation: *R*_*G*_ = 7, *R*_*C*_ = 5, *R*_*S*_ = 6, *C*_*G*_ = 6, *C*_*C*_ = 8, *C*_*S*_ = 8, *L*_*G*_ = 3, *L*_*C*_ = 1, *L*_*S*_ = 2, Δ*R*_*G*_ = 4, Δ*R*_*C*_ = 2, Δ*R*_*S*_ = 3, α = 0.3, β = 0.4, γ = 0.1. Let the initial strategy probability be (*x*_0_, *y*_0_, *z*_0_) = (0.5, 0.5, 0.5). The evolutionary trajectory of the game based on the initial value is depicted in Figure 4.

Based on Figure 4, under the given initial parameter settings, the strategic choices of the government and sports Organization exhibit continuous fluctuations over time. In contrast, the community demonstrates a clear preference for the passive strategy, with its active strategy probability rapidly declining and stabilizing near zero. This divergence in strategic adaptation underscores the asymmetric incentives and cost-benefit structures among the three stakeholders, which may hinder the emergence of a stable, fully cooperative equilibrium in the absence of targeted policy interventions.

To further understand how different policy interventions affect the evolution of community sports governance, we conduct a series of parameter sensitivity analyses focusing on subsidy levels (α), performance evaluation discount coefficient (β), and information platform effectiveness (γ).

### 3.1 Impact of subsidy levels

**Figure 5:**
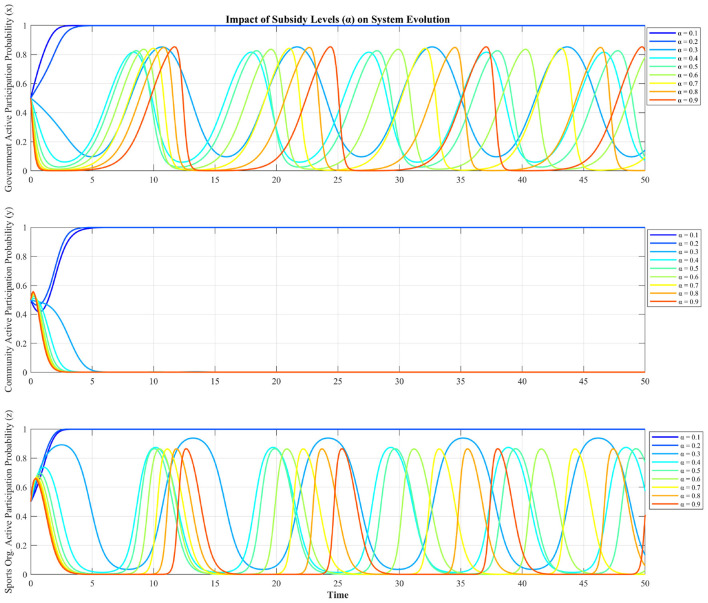
Impact of subsidy levels on system evolution.

We first examine how different subsidy levels (α) affect the system evolution. Figure 5 demonstrates the differential impact of the government subsidy rate (α) on the three stakeholders. The active participation probability of the government (*x*) and the sports organization (*z*) exhibits persistent, high-amplitude oscillations over time, with higher α values generally correlating with increased volatility. In stark contrast, the community's active participation (*y*) rapidly decays and converges to near-zero for all subsidy levels, indicating complete strategic withdrawal.

The simulation demonstrates a non-linear relationship between subsidy levels (α) and tripartite collaboration outcomes. This reveals a critical limitation of fiscal subsidies as a standalone policy. Under low subsidy regimes (α < 0.3), cooperation is driven primarily by synergistic benefits, as minimal fiscal incentives maintain manageable costs for the government while motivating community and sports organizations to engage actively for mutual gains. In contrast, high subsidy regimes trigger systemic collapse into non-cooperation: excessive subsidies impose unsustainable fiscal burdens on the government, discouraging proactive involvement, while simultaneously creating a “subsidy dependency” among community and sports organizations, where high transfers reduce the marginal utility of synergistic efforts, fostering free-riding behavior. This inverse effect underscores that subsidies function as a double-edged sword, with their efficacy constrained by critical thresholds. This reflects that the incentive must be moderate, with the goal of activating endogenous cooperative power, rather than simply “spending money to buy cooperation”.

### 3.2 Impact of performance evaluation discount coefficient

**Figure 6:**
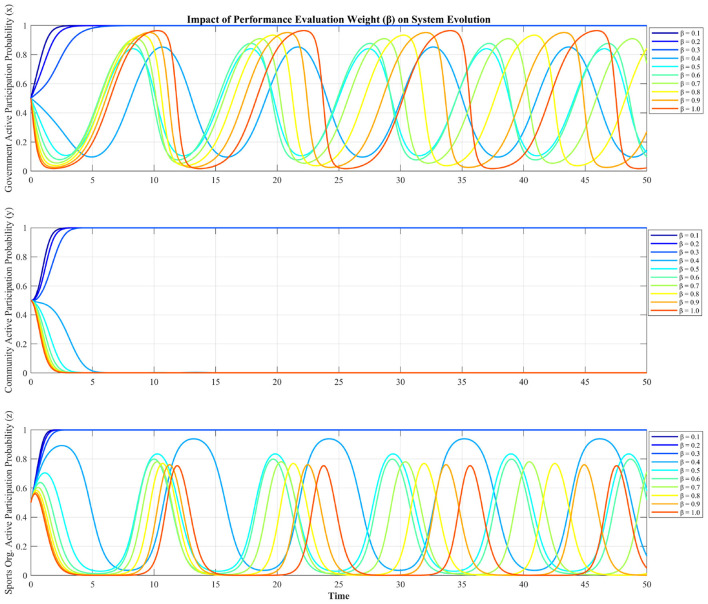
Impact of performance evaluation discount coefficient on system evolution.

Next, we analyze how performance evaluation discount coefficient (β) affects system evolution (see Figure 6). It can be seen from Figure 6 that the performance evaluation discount coefficient (β) exerts a sharp, threshold-dependent influence on the community's strategic choices, producing distinct shifts in collective behavior as (β) crosses critical values. A critical bifurcation occurs at β = 0.3. For β ≤ 0.3, the community's participation probability (*y*) stabilizes at a high level (*y* → 1). For β≥0.4, it collapses to near zero (*y* → 0). This abrupt transition indicates that the community's strategy is exquisitely sensitive to the government's “free-riding” propensity: when the government can appropriate a large share (β≥0.4) of the collaborative outcomes without active involvement, the community withdraws entirely.

The simulation demonstrates the performance evaluation discount coefficient β acts as a “strategic switch” for the stakeholders engagement. This non-linear response highlights the delicate balance required in performance-based governance. Namely, a high level of administrative pressure (low β) effectively eliminates the temptation for government free-riding, thereby fostering a stable, fully cooperative environment. Conversely, a relaxation of this pressure (high β) introduces strategic ambiguity, leading to persistent oscillations where no single strategy can maintain dominance. While excessive top-down pressure might be undesirable in some contexts, a certain degree of “constructive pressure” (i.e., a sufficiently low β) is essential to align the government's incentives with the broader goal of collaborative governance. The findings suggest that policymakers must carefully calibrate performance evaluation mechanisms to ensure they provide sufficient impetus for sustained proactive engagement.

### 3.3 Impact of information platform effectiveness

**Figure 7:**
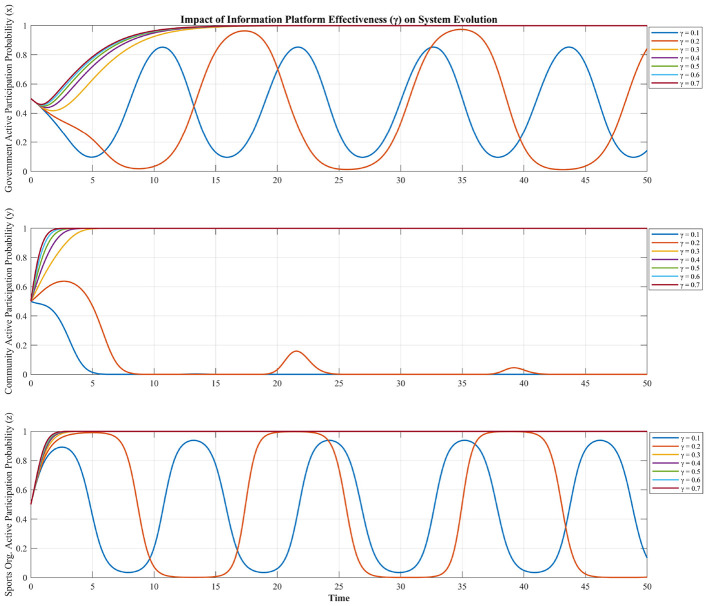
Impact of information platform effectiveness on system evolution.

We also examine how information platform effectiveness (γ) affects system evolution (see Figure 7). From Figure 7, as γ increases from 0.1 to 0.7, the three stakeholders' participation probability not only rises more rapidly but also reaches a higher steady state. Higher γ values correspond to steeper initial growth and a more stable convergence toward active participation (*y* → 1). This monotonic positive relationship demonstrates that reducing informational frictions directly enhances the stakeholders' perceived benefits of collaboration, making active engagement a dominant strategy.

The simulation demonstrates the information platform parameter γ functions as a “participation amplifier” for three stakeholders. This analysis underscores the profound impact of information platform effectiveness as a digital catalyst for fostering stable, collaborative governance. Unlike fiscal incentives or administrative pressures, which can, under certain conditions, induce strategic oscillations, a well-functioning information platform consistently reduces coordination costs, particularly for resource-constrained community organizations. This reduction acts as a powerful positive externality, incentivizing active participation across all stakeholders and effectively “locking in” a cooperative equilibrium. This result highlights that investments in digital governance infrastructure (γ) are not merely about efficiency gains but are fundamental to achieving systemic stability and sustained multi-agent collaboration. For policymakers, this suggests that alongside traditional incentives, the strategic deployment of technological solutions can be a highly effective pathway to cultivating robust and enduring partnerships in complex governance challenges. This finding is strongly supported by the literature on digital governance, which posits that digital tools reduce coordination costs and mitigate information asymmetry among decentralized actors (37).

### 3.4 Impact of policy combinations

**Figure 8:**
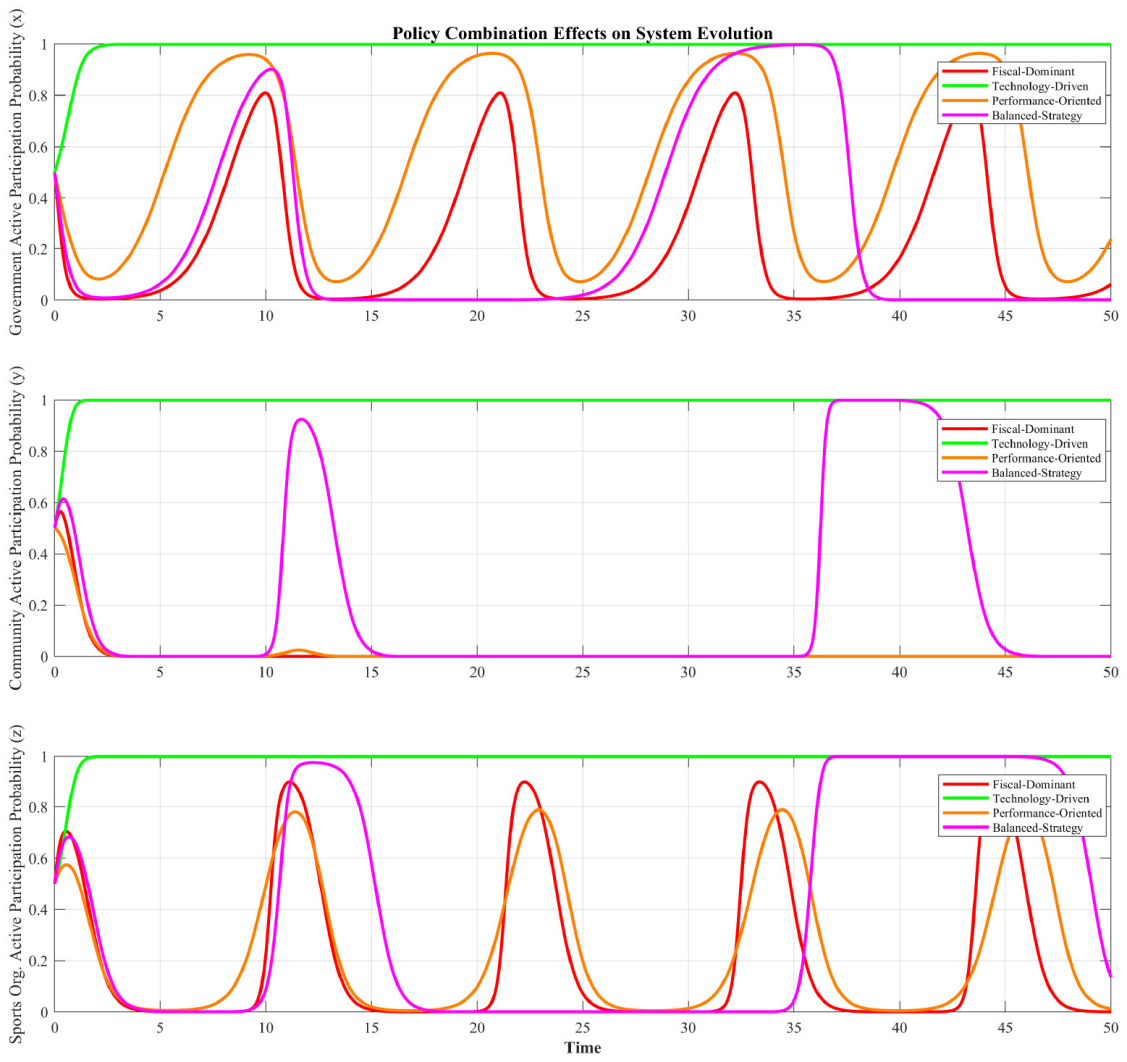
Impact of policy combinations on system evolution.

Finally, we analyze how different combinations of policy instruments affect system evolution. Figure 8 compares four distinct policy combinations: Fiscal-Dominant (α = 0.8, β = 0.2, γ = 0.1), Technology-Driven (α = 0.2, β = 0.3, γ = 0.7), Low-Pressure (α = 0.2, β = 0.8, γ = 0.2), and Balanced-Strategy approaches (α = 0.5, β = 0.5, γ = 0.5). Each policy combination reflects a distinct governance logic. The Fiscal-Dominant strategy relies on high fiscal inputs combined with stringent oversight (low β), representing a resource-intensive, incentive-based mode. The Technology-Driven combination features a dominant γ, highlighting innovation-led governance, with restrained fiscal and performance pressures. The Low-Pressure strategy operates with minimal subsidies and technology under lenient supervision (high β), reflecting a passive governance mode with attenuated accountability. Lastly, the Balanced-Strategy maintains equal weighting across all three parameters, seeking integrated but potentially diluted policy effects. From Figure 8, the “Technology-Driven” strategy achieves the highest and most stable participation level for all participants, followed by the “Balanced-Strategy” which shows considerable volatility. However, the “Fiscal-Dominant” and “Low-Pressure” strategies yield lower and more erratic trajectories for the community. This indicates that a policy mix emphasizing technological empowerment (high γ) is most effective in securing the community's commitment, whereas over-reliance on subsidies or lenient oversight alone leads to sub-optimal and unstable outcomes.

The policy combination analysis demonstrates how different strategic approaches to sustainable community sports governance perform under integrated policy frameworks. The analysis of policy combinations confirms that the effectiveness of the policy mix is highly dependent on the dominant instrument and the resulting strategic robustness (39). Moreover, the comparative analysis reveals that technological empowerment is the cornerstone of sustainable tripartite cooperation. This demonstrates that strategic investment in digital infrastructure to reduce coordination costs (high γ) can be a more powerful and stabilizing force than solely relying on high fiscal incentives or administrative pressure. When combined with moderate subsidies and performance incentives, it generates a synergistic effect that stabilizes the all participants' participation, especially for the community. In contrast, policies that neglect informational infrastructure fail to provide the coordination clarity needed for long-term engagement. The findings advocate for a policy portfolio that prioritizes platform effectiveness as the foundational enabler, then calibrates financial and regulatory instruments to reinforce—not replace—the trust and transparency created by the information platform.

## 4 Discussion

### 4.1 Theoretical implications

We contribute to the theoretical understanding of community sports governance in several important ways. First, the study extends the application of evolutionary game theory to tripartite collaborative governance, moving beyond the bilateral frameworks that dominate existing literature. By modeling the complex interactions among local government, communities, and sports organizations, the study provides a more comprehensive understanding of the dynamic mechanisms underlying community sports governance. Furthermore, our study identifies a “policy trap” where moderate incentives fail to achieve a stable equilibrium, leading instead to persistent strategic uncertainty.

Second, the study explicitly models the political performance pressures inherent in the Chinese local government. By incorporating the “pressure-type system” into the local government's payoff function, we provide a mechanism-based explanation for the risk of “goal displacement”—a common pathology in Chinese public administration—where excessive pressure (β) causes local actors to substitute the goal of collaborative governance with the goal of political risk minimization. In public health governance, top-down pressures can divert resources from community-based health programs to formalistic compliance, undermining long-term health equity. This offers a more nuanced understanding of the complexities of local governance practice in China.

Third, the study advances our understanding of policy instrument mix complexity by revealing the non-linear and synergistic effects of policy interventions on governance system evolution. Unlike conventional studies that focus on static, linear effects (40), our model identifies critical threshold effects across all policy parameters (α, β, γ). Specifically, the simulation results demonstrate that the effectiveness of policy instruments is threshold-driven rather than incremental, with direct implications for public health outcomes. By explicitly modeling γ as a reducer of coordination costs for community organizations, we demonstrate that technological intervention can be a more robust stabilizer than fiscal or administrative measures.

Furthermore, our research contributes to the theoretical literature on institutional change in sport governance by demonstrating how evolutionary processes can lead to transformative governance innovations despite path dependencies and institutional inertia. Differing from (41)'s institutional analysis of continuity and change in sport organizations, the study extends this framework to account for the dynamic interactions among multiple stakeholders in complex governance systems. The identification of critical thresholds and tipping points in governance evolution offers a more nuanced understanding of how incremental policy changes can ultimately lead to systemic transformation.

### 4.2 Policy implications

Based on the analysis of the tripartite evolutionary game model, we derive four specific and actionable policy recommendations that address the non-linear dynamics and institutional complexities of Chinese community sports governance. These recommendations are structured to optimize the policy instrument mix (α, β, γ) and ensure a robust transition to a collaborative equilibrium.

(1) Transition from “subsidy-heavy” to “technology-enabled” governance. Policymakers should shift their focus from purely increasing fiscal subsidies (α) to investing in robust digital governance infrastructure (γ). As shown in the Technology-Driven scenario, high platform effectiveness can achieve stable cooperation even with lower fiscal outlays. Developing integrated digital platforms that streamline venue booking, activity coordination, and resident outreach can fundamentally lower the “entry barrier” for community organizations, making collaboration a default rather than a difficult choice.

(2) Calibrate “constructive pressure” through dynamic evaluation. Rather than a one-size-fits-all performance metric, superior governments should implement a dynamic evaluation system that adjusts the “discount coefficient” (β) based on local conditions. The evaluation should move beyond simple “output” metrics to include “process” and “synergy” indicators. This ensures that local governments remain proactively engaged not just to avoid penalties, but to secure long-term governance benefits.

(3) Avoid the “moderation trap” with targeted policy portfolios. The failure of the “Balanced-Strategy” approach suggests that a “little bit of everything” is often insufficient. Policy-makers should instead adopt a “lead instrument” strategy. For instance, in resource-scarce regions, a “Technology-Driven” approach should be the priority, while in regions with high administrative capacity, a “Low-Pressure” approach with strict accountability might be more effective. The key is to ensure that at least one policy instrument reaches the critical value necessary to break the cycle of strategic oscillation.

### 4.3 Limitations and future directions

Despite its contributions, this study has several limitations that suggest directions for future research. First, the model assumes a degree of rationality and information symmetry that may not fully reflect the complexities of real-world governance. Future research could incorporate greater heterogeneity, distinguishing between different levels of government (central, provincial, municipal), types of community organizations (residents' committees, social organizations, volunteer groups), and categories of sports organizations (professional associations, commercial enterprises, nonprofit clubs). This more nuanced approach would align with Nagel et al. (42)'s multi-level framework for analyzing forms, causes, and consequences of professionalization in sport federations. Second, the empirical validation of the model relies on numerical simulations rather than field experiments or longitudinal data. Future research could test the model's predictions through empirical studies, potentially using pilot policy interventions to assess their impact on stakeholder behaviors and governance outcomes. This approach would address Sam et al. (43)'s call for more evidence-based approaches to sport governance reforms, moving beyond theoretical models to practical implementation and evaluation. Finally, future research could examine the transferability of the proposed governance framework to other public service domains facing similar multi-stakeholder coordination challenges, such as community healthcare, environmental protection, and cultural development. This comparative approach would contribute to broader theoretical discussions about collaborative governance in complex policy domains (44), while providing practical insights for integrated community development initiatives that span multiple sectors and policy areas.

## 5 Conclusions

This study develops an asymmetric tripartite evolutionary game model to examine the dynamic mechanisms underlying community sports governance in China, with a particular focus on the non-linear effects of a policy instrument mix (α, β, γ). The primary contribution of this research lies in identifying the “oscillation traps” associated with moderate policy interventions and highlighting the transformative potential of digital platforms in stabilizing multi-agent cooperation. From a public health perspective, these results suggest that the consistent availability of community sports resources—a prerequisite for long-term health behavior change—is best secured through policies that prioritize technological infrastructure and clear administrative accountability. Our major results are as follows.

First, enhancing information platform effectiveness (γ) is the most robust strategy for overcoming the coordination barriers faced by community organizations, thereby ensuring the sustainable delivery of health-promoting sports activities. Second, maintaining a certain level of “constructive pressure” through performance evaluation (β) is necessary to prevent government free-riding and ensure proactive support for public health initiatives. Finally, policymakers must move beyond isolated interventions and adopt integrated policy portfolios that leverage technological catalysts to break the cycle of strategic uncertainty.

These results offer valuable policy implications for promoting a transition from government-centric to a robust collaborative governance model. Ultimately, optimizing the governance of community sports is a vital strategy for addressing the growing burden of non-communicable diseases and improving the overall well-being of urban populations.

## Data Availability

The original contributions presented in the study are included in the article/supplementary material, further inquiries can be directed to the corresponding author.
